# MFsim—an open Java all-in-one rich-client simulation environment for mesoscopic simulation

**DOI:** 10.1186/s13321-020-00432-9

**Published:** 2020-05-01

**Authors:** Karina van den Broek, Mirco Daniel, Matthias Epple, Jan-Mathis Hein, Hubert Kuhn, Stefan Neumann, Andreas Truszkowski, Achim Zielesny

**Affiliations:** 1 Inorganic Chemistry and Center for Nanointegration Duisburg-Essen (CeNIDE), University of Duisburg-Essen, Essen, Germany; 2 Institute for Bioinformatics and Chemoinformatics, Westphalian University of Applied Sciences, August-Schmidt-Ring 10, 45665 Recklinghausen, Germany; 3 CAM-D Technologies GmbH, Solingen, Germany; 4 GNWI – Gesellschaft für naturwissenschaftliche Informatik mbH, Dortmund, Germany; 5 Yara Deutschland, Dülmen, Germany

**Keywords:** Molecular simulation, Mesoscopic simulation, Rich-client, Model-view-controller, MVC, Pattern, Graphical user interface, GUI, Dissipative particle dynamics, DPD, Molecular fragment dynamics, MFD, PDB parser

## Abstract

MFsim is an open Java all-in-one rich-client computing environment for mesoscopic simulation with Jdpd as its default simulation kernel for Molecular Fragment (Dissipative Particle) Dynamics. The new environment comprises the complete preparation-simulation–evaluation triad of a mesoscopic simulation task and especially enables biomolecular simulation tasks with peptides and proteins. Productive highlights are a SPICES molecular structure editor, a PDB-to-SPICES parser for particle-based peptide/protein representations, a support of polymer definitions, a compartment editor for complex simulation box start configurations, interactive and flexible simulation box views including analytics, simulation movie generation or animated diagrams. As an open project, MFsim allows for customized extensions for different fields of research. 
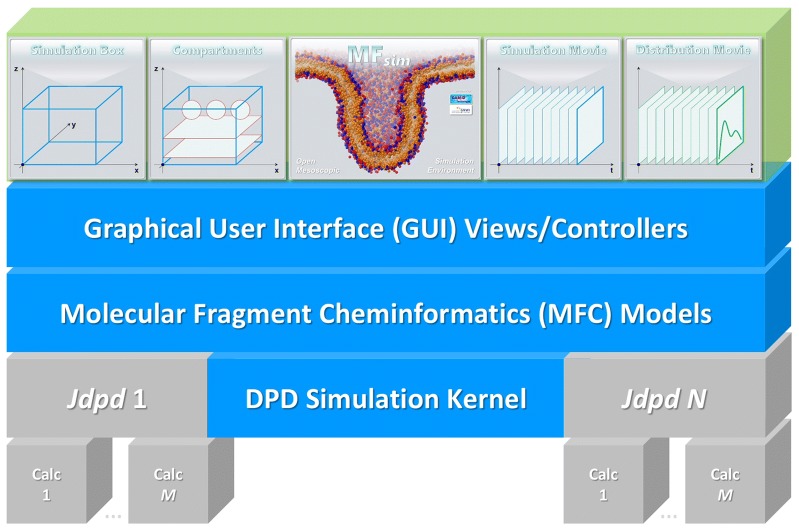

## Introduction

A molecular simulation workflow comprises three successive steps: The definition of a simulation job with all necessary input information (preparation step), the approximate numerical integration of the equations of motion (the actual simulation step) and the analysis of the simulation record with all calculated results (evaluation step). A computational all-in-one rich-client environment aims at supporting this triad in a unified and comprehensive manner to allow for productive applicability with minimum effort, minimized training periods and usability without programming skills. An additional goal is the prevention of common problems like inadequate or ill-defined parameter settings by extensively safeguarding simulation job definitions and operations. In general, these desirable features require a highly integrated monolithic architecture: Its pre-defined optimized workflows are comfortable and fast but inflexible in comparison with scripting or pipelining-workflow approaches. For the latter the implementation of a new feature may be achieved within minutes whereas its rich-client realization may involve days and weeks of complex software development. Thus, the different approaches have their intrinsic strengths and weaknesses.

Particle-based mesoscopic simulation investigates supramolecular phenomena at the nanometer length and microsecond time scale for large interacting physical ensembles representing millions of atoms. Dissipative Particle Dynamics (DPD) is a mesoscopic simulation technique for isothermal complex fluids and soft matter systems which satisfies Galilean invariance and isotropy, conserves mass and momentum and achieves a rigorous sampling of the canonical NVT ensemble due to soft particle pair potentials that diminish molecular entanglements or caging effects. DPD is expected to show correct hydrodynamic behavior and to obey the Navier–Stokes equations. DPD particle trajectories are guided by Newton’s equation of motion where the total force on a particle exerted by other particles consists of a conservative, a dissipative (frictional) and a random part. The opposing dissipative and random forces depend on each other and act as a thermostat conserving the total momentum and introducing Brownian motion into the system. The conservative forces comprise soft DPD particle repulsions as well as possible harmonic springs between bonded and electrostatic interactions between charged particles [1–5]. DPD particles in general may be arbitrarily defined as “fluid packets”. Molecular Fragment Dynamics (MFD) is a “bottom-up” DPD variant which chooses the particles to be small “fragment molecules” of molar mass in the order of 100 Da where larger molecules are composed of adequate smaller “fragment molecule” particles that are bonded by harmonic springs to mimic covalent connectivities and spatial 3D conformations [5–12].

The new MFsim project provides (to our knowledge) the first open Java all-in-one rich-client mesoscopic simulation environment and complements the few available commercial systems [13, 14] with specific support for biomolecular applications containing peptides and proteins. By default, MFsim is integrated with the Jdpd simulation kernel, an open MFD Java code [15, 16]. As an open approach, MFsim is not restricted to a specific mesoscopic simulation engine but may be customized to communicate with any particle-based simulation code [17–24]: Appendix 3 outlines the corresponding simulation kernel integration details.

Usage of MFsim does not require programming skills and supports the complete preparation-simulation–evaluation triad of a mesoscopic simulation task. It comprises features like a SPICES line notation [25, 26] based chemical structure editor, a PDB file parser [27] for particle-based peptide/protein representations, a peptide and protein editor, support of polymer definitions, a compartment editor for complex molecular start configurations and interactive simulation box views including analytics, on-the-fly movie generation and animated diagrams. Parameter settings are supported by reusable schemata and filtered bulk operations, inter-parameter dependencies are controlled by directed internal update cascades to avoid ill-defined settings. MFsim parallelizes operations to effectively exploit multi-core processor hardware.

## Implementation

**Fig. 1:**
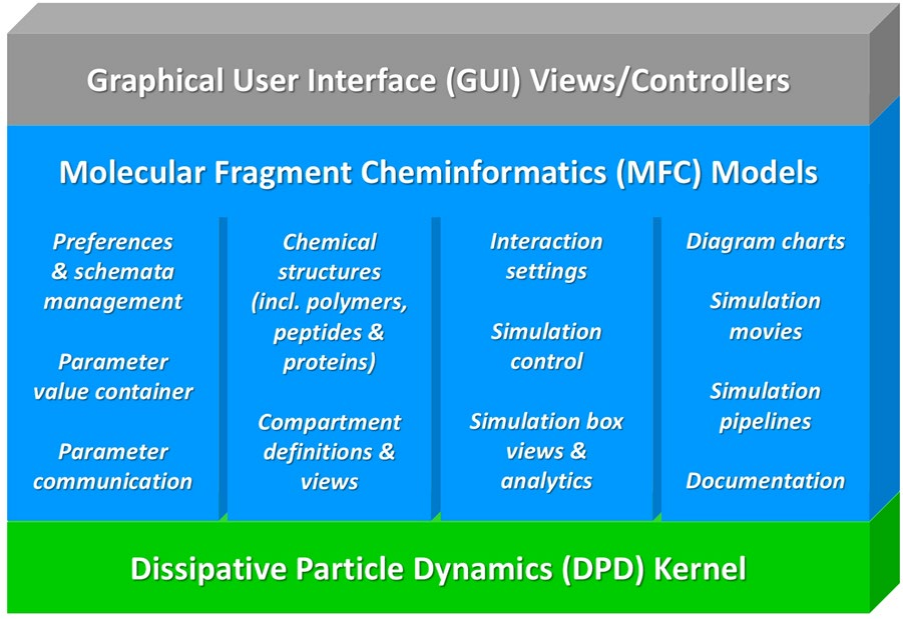
MFsim architecture with underlying MVC pattern: Graphical user interface (GUI) views/controllers communicate with a layer of Molecular Fragment Cheminformatics (MFC) models that provide all core functions where the MFC layer itself controls the DPD simulation kernel layer

The object-oriented Java architecture (see Fig. 1) follows a Model-View-Controller (MVC) pattern [28]: Graphical user interface (GUI) view classes (based on the Swing GUI Toolkit [29] of the Java platform [30]) are governed by corresponding controller classes (*gui* packages *control, dialog* and *main*). The controllers communicate with a layer of Molecular Fragment Cheminformatics (MFC) models (*model* packages *changeNotification*, *graphics*, *jmolViewer*, *job*, *message*, *particle*, *particleStructure*, *peptide*, *preference*, *util* and *valueItem*) that provide all core functions where the MFC layer itself controls the particle simulation kernel (Jdpd as a default).

**Fig. 2:**
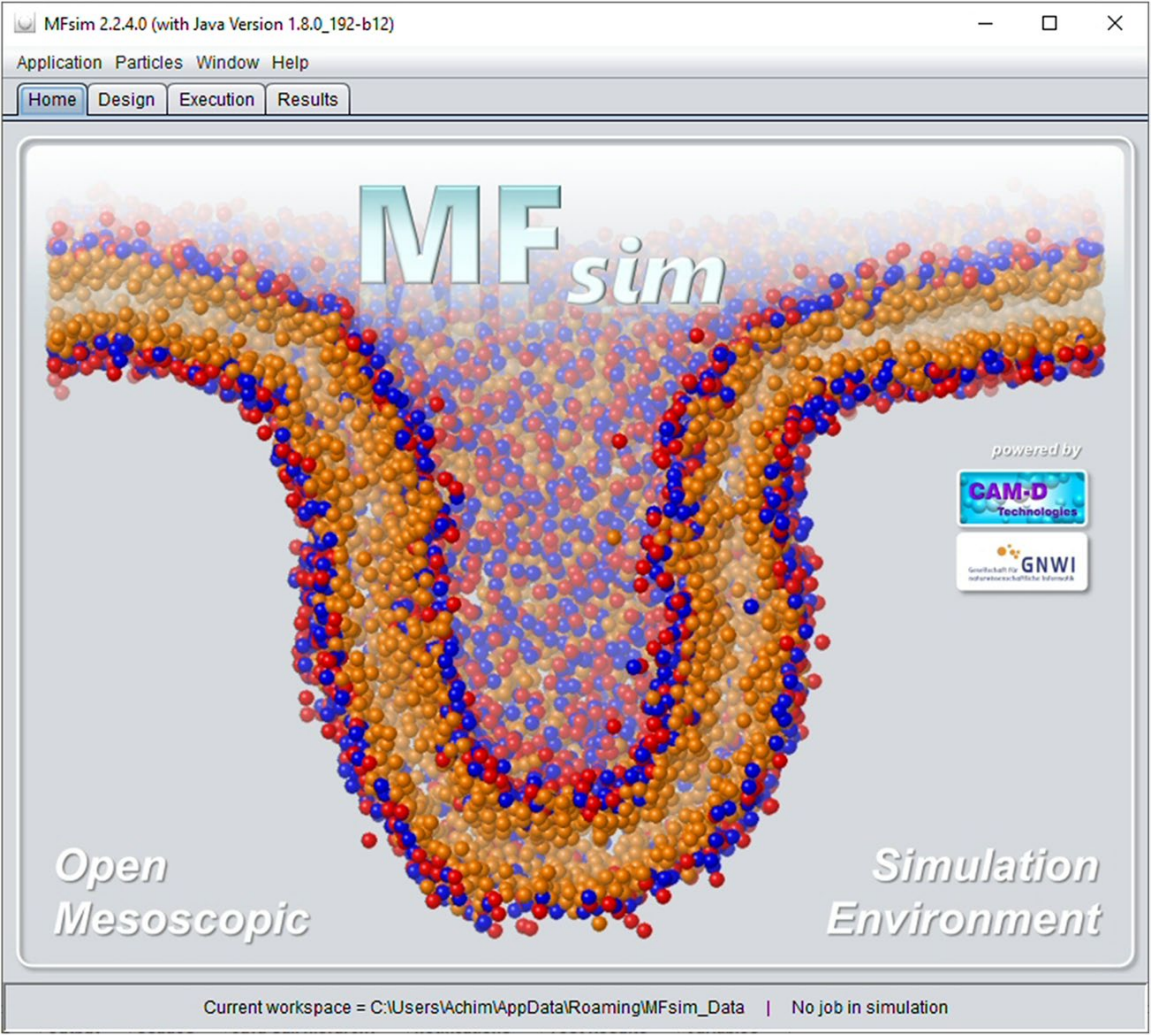
Basic MFsim GUI frame with three management tabs for simulation job *Design*, job *Execution* and job *Results* evaluation operating on a defined workspace directory (see status line at the bottom). The displayed *Home* tab depicts graphical project information and links to the MFsim GitHub repository

The basic GUI frame is organized by three management tabs for simulation job *Design*, job *Execution* and job *Results* evaluation that operate on a defined workspace directory of the file system (see Fig. 2): This workspace directory contains a *JobInputs* folder (where each subfolder corresponds to a single *Job Input* definition) and a *JobResults* folder (with subfolders corresponding to *Job Result* instances). During job execution a temporary directory is used for file storage which may be located on an fast hardware/memory device for maximum performance. All specific GUI functions are provided by successive levels of modal dialogs that can be opened above the basic GUI frame.

MFsim automatically logs internal problems to support the detection of possibly subtle errors which are likely to occur in complex architectures with hundred thousands of code lines. The log file entries may be viewed via menu entry *Help/MFsim log/Browse* of the basic GUI frame.

Details about internal data objects, preferences and convenient re-usable settings are comprised in Appendix 2.

MFsim supports concurrent calculations to exploit the capabilities of multi-core processors for performance improvements that (almost) linearly scale with the number of available processor cores (and additionally benefit from technologies like Hyper-Threading). To arrive at an overall optimum balance the global preference section *Parallel computing* provides options for fine-tuning of concurrent operations. As an example the number of parallel calculation threads for force calculations of the (default Jdpd) particle simulation engine may be specified in combination with the number of concurrent jobs in simulation to achieve an adequate workload of an octa-core processor with 2 concurrent simulations and 4 force calculation threads each—whereas for a maximum single-job performance the number of force calculation threads could be increased to 8 with the number of parallel simulations decreased to 1. Additional parallel computing options address the number of concurrent particle position writers for output file creation, the minimum number of simulation box cells and particle–particle bonds for parallelization (to avoid ineffective parallelization efforts due to the necessary computational overhead) and the number of concurrent graphical simulation box slice and diagram generators (abbreviated slicers – their number should at best correspond to the number of available processor threads including Hyper-Threading).

Another performance related feature is global caching: SPICES line notations (class *SpicesPool* of package *model.particleStructure*) and PDB file related protein definitions (class *PdbToDpdPool* of package *model.peptide*) are kept in memory to speed-up chemical structure related operations by avoidance of expensive reevaluations during calculations. The size of the SPICES and protein cache may be inspected via menu entry *Application/Cache/Show* of the basic GUI frame. In-memory caching also improves the performance of simulation box slice image related operations: *Preference/Simulation box/Slicer graphics/Image storage* in the global preferences dialog may be used to activate the *Memory uncompressed* (fastest) or *compressed* (smallest) image cache, otherwise slice images are stored as image files which is comparably slow.

MFsim slicer graphics is realized with Java2D [31]. 3D displays of the simulation box are parallel projections that allow for versatile through-space measurements (but appear somewhat distorted compared to central projections). The spatial 3D impression is additionally supported by a configurable fog generation. Simulation movie generation is realized by merging box images into a movie clip utilizing the open FFmpeg software [32].

Since there is no unique particle set for mesoscopic simulation, a specific field of research requires a specific particle set—a situation that is similar to Molecular Mechanics/Dynamics with different force fields and specific atom types. All mandatory particle information and particle–particle interactions must be provided by a particle set text file where particle definitions are to be described in mandatory section *[Particle description]*, particle–particle repulsions in mandatory section *[Particle interactions]* and peptide/protein decomposition related particle information in optional section *[Amino acids]*. MFsim comes with *ParticleSet_H2O.txt*, a minimal single-particle set for test purposes, and *ParticleSet_AA_V02.txt*, a basic biomolecular particle set for an approximate fragmentation of phospholipids and peptide/proteins. The latter is based on the particle data in [24] with rescaled molecular volumes and the water molecule being the smallest particle of volume 30 Å^3^. MFsim contains some functions for particle set manipulation (e.g. particle duplication, see menu *Particles* of the basic GUI frame) and supports the automated update of a *Job Input* definition for a new particle set.

Last but not least—as an open project itself, MFsim uses several other open libraries: Apache Commons IO [33] /Lang [34] /RNG [35], BioJava [36, 37], FFmpeg (currently with the Windows OS only), GraphStream [38], Jama [39], JCommon [40], Jdpd, JDOM [41], JFreeChart [42], Jmol [43], PCG [44, 45], SPICES [26] and Vecmath [46].

## Results and discussion

The MFsim simulation system aims at supporting the complete preparation-simulation–evaluation triad in form of an integrated all-in-one workflow which is sketched step-by-step in the following paragraphs.

### Simulation job design

The simulation *Design* tab of the basic GUI frame manages all *Job Input* related operations. It comprises an optionally filtered list of all available *Job Input* definitions of the specified workspace where a single *Job Input* definition may be viewed, edited, re-used (as a start for a new definition), removed, imported from an archive file or itself archived to a file (e.g. for exchange purposes). For a new *Job Input* definition, a corresponding modal dialog is opened above the basic GUI frame.

**Fig. 3:**
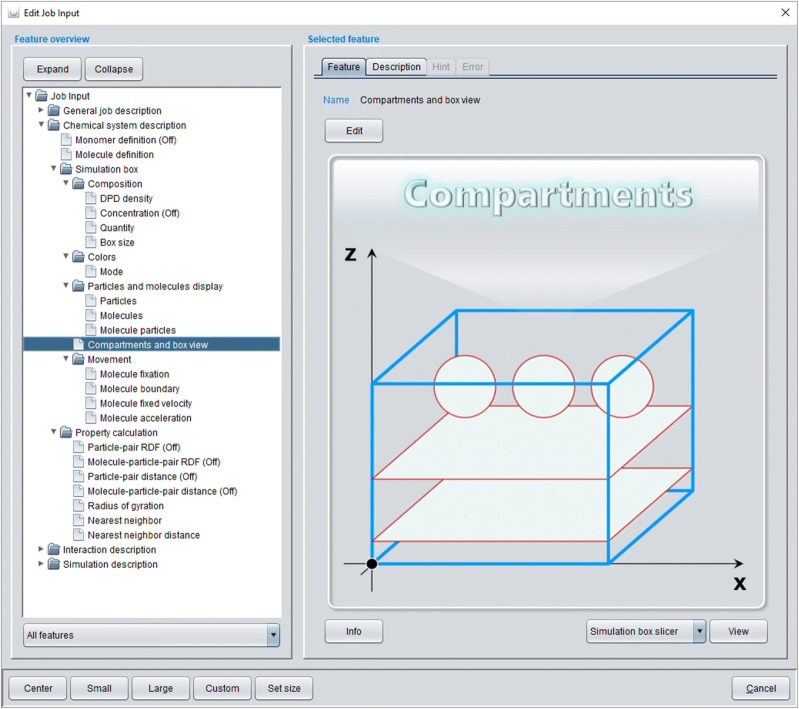
Modal dialog for a *Job Input* definition with main sections *General job description* (collapsed), *Chemical system description* (expanded), *Interaction description* (collapsed) and *Simulation description* (collapsed). Feature *Compartments and box view* of subsection *Simulation box* in main section *Chemical system description* is selected

A new *Job Input* definition is already a complete and valid simulation job definition with a single particle type (the water particle). The *Job Input* features have to be defined in a top-down manner where a change in a higher-level feature leads to an immediate update of subordinate features in an adequate manner—e.g. a change in the number of particles via the *Quantity* feature automatically changes the subordinate *Box size* feature to be consistent with the new higher-level *Quantity* settings according to the even higher *DPD density* setting. These successive top-down updates (which are realized by directed *ValueItem* update cascades, see class *UtilityJobUpdate* in package *model.job* as well as Appendix 2) alleviate error-free *Job Input* definitions with an overall logical integrity. A complete *Job Input* definition is organized in form of a feature tree that consists of four main sections (see Fig. 3): The *General job description*, the *Chemical system description*, the *Interaction description* and the *Simulation description*. Every single *Job Input* feature contains a local *Description*, a *Hint* and an *Error* tab: The *Description* tab provides a descriptive outline of the feature in question and summarizes its possible settings. The *Hint* tab informs about possible shortcomings of the current setting (e.g. possibly unwanted identical colors for different molecules so that the molecules cannot be distinguished in a simulation box display) whereas an activated *Error* tab signals a severe problem which has to be resolved (an erroneous *Job Input* definition is not allowed to be executed). Since the list of *Job Input* features should exploit the range of capabilities of the underlying particle simulation kernel the current MFsim *Job Input* feature set especially addresses the default Jdpd simulation kernel (see class *JdpdValueItemDefinition* in package *model.job*). For other particle simulation kernels the feature set would have to be customized accordingly (see Appendix 3).

The *General job description* comprises job related information like a *Description* line or a creation *Timestamp*.

**Fig. 4:**
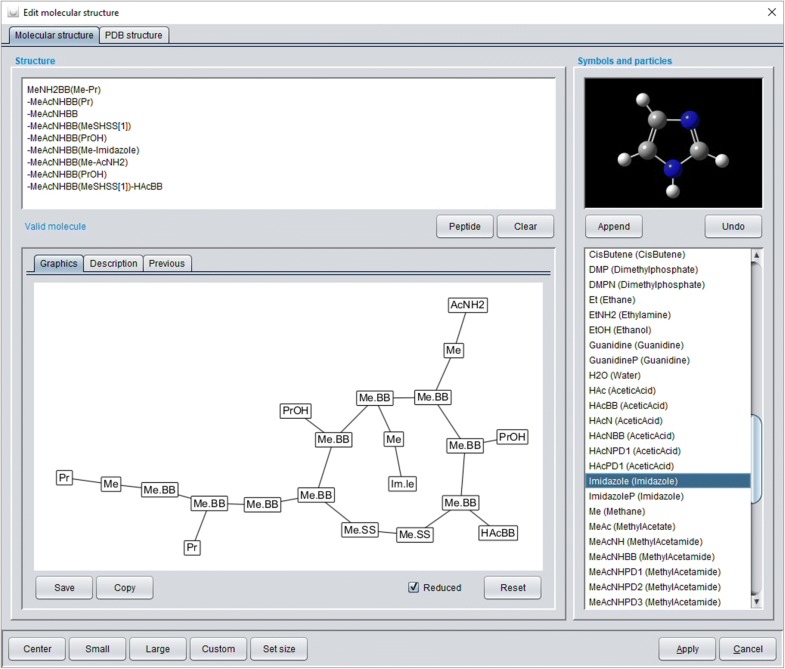
*Molecular structure* tab of the modal structure editor dialog: The SPICES line notation (upper left) of a cyclic peptide was prepared with the modal peptide dialog (available via the active *Peptide* button) thus the line notation is automatically split into several lines for each amino acid to alleviate comprehension. The notation is valid (confirmed by the *Valid molecule* label) and its cyclic topological structure is displayed below. Available structure elements (particles, brackets, tags etc.) are listed on the right

The *Chemical system description* addresses all features that characterize the chemical ensemble to be simulated. *Monomer definition* and *Molecule definition* are realized with chemical structure editors plus specific editors for peptides and proteins. The editor for monomers and molecular structures is based on the SPICES and GraphStream libraries [25] and supports the input of a SPICES line notation. A SPICES string can be manually coded (or composed by clicking on available structure elements like particles, brackets or tags) and is immediately parsed with its manipulative topological structure displayed below (for a valid definition)—thus the input of an illegal SPICES line notation is (hopefully) impossible. If a particle set with amino acids definition is chosen the *PDB structure* tab and the *Peptide* button are activated. The additional modal peptide dialog allows for the input of a one-letter-code peptide sequence (manually or by clicking on the available amino acids and structure elements like disulfide bridges, charges etc.) that is afterwards converted to a SPICES line notation in the structure editor (see Fig. 4).

**Fig. 5:**
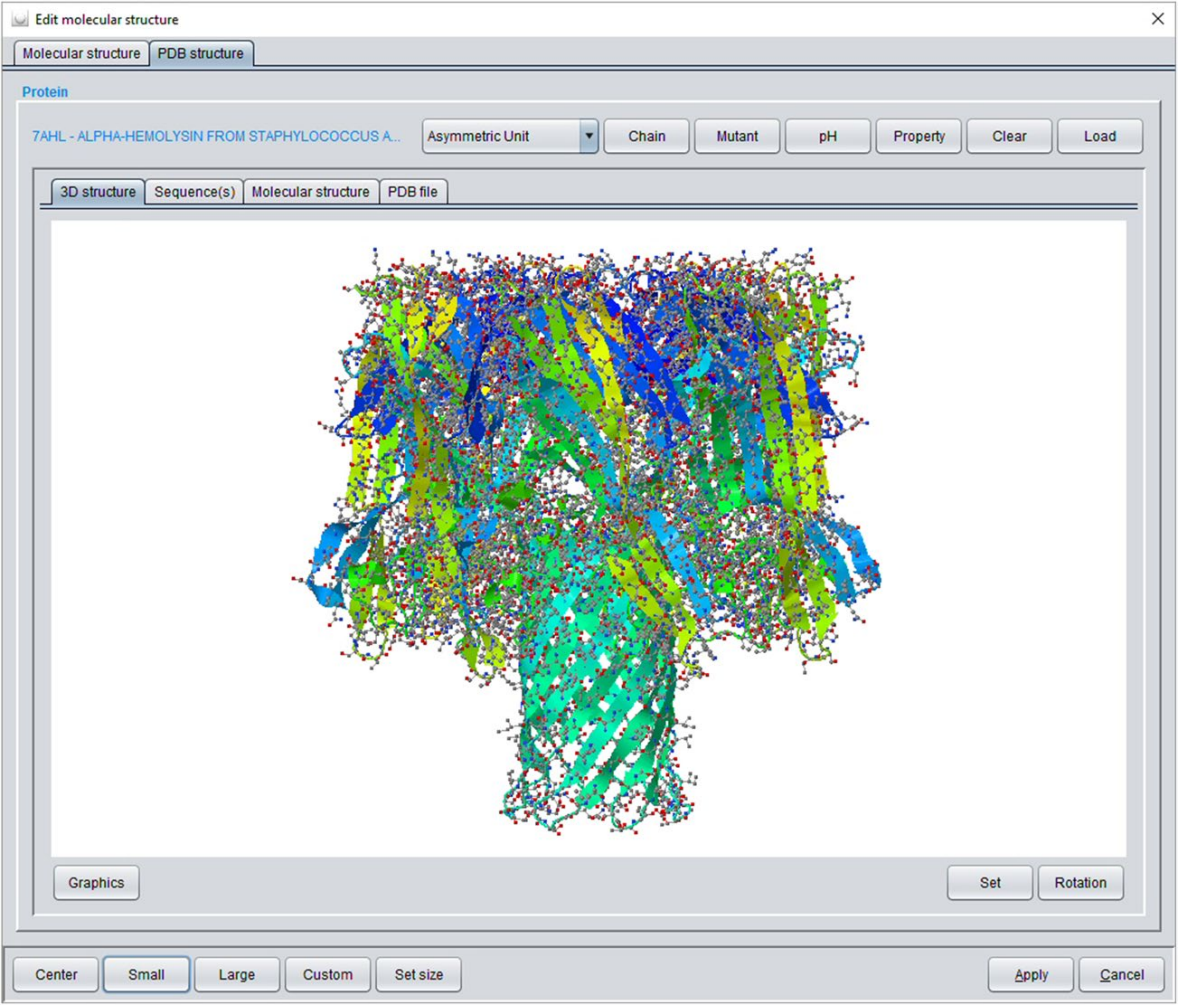
*PDB structure* tab of the modal structure editor dialog: A PDB file can be loaded with automated conversion to a SPICES line notation. Different tabs show the rendered protein structure, its amino acid sequence, the corresponding SPICES line notation and the text of the PDB file. Several additional functions and dialogs are available at the top (see text)

For biomolecular simulations MFsim provides a PDB-to-SPICES parser which is available via the *PDB structure* tab (see Fig. 5). An opened PDB file is rendered with the Jmol library and internally evaluated with the BioJava library to create a corresponding SPICES line notation. Different biological assemblies may be chosen from the PDB definition via a drop-down box. The modal *Chain* dialog may be used to exclude protein chains and to a priori assign a segment to an amino acid according to its protein chain. The modal *Mutant* dialog allows the specific change of amino acids to create in silico mutants (with the same 3D structure of the backbone chain). A pH value may be set with the modal *pH* dialog to charge corresponding particles. The modal *Property* dialog offers a number of settings for the amino acids of all chains: Protein backbone particles may be replaced by probe particles with identical physical properties but different labels for e.g. selective display of specific amino acids of a chain. In addition, a status and a segment may be defined for each amino acid backbone particle which can be used for intra-protein force assignments in order to achieve specific control concerning the flexibility or stiffness of the protein backbone (whereas amino acid side chain particles are always flexible). The complete protein information is encapsulated in a *PdbToDpd* object (package *model.peptide*) which is globally stored in the protein cache for inexpensive reuse in protein related calculations. A *PdbToDpd* object also comprises an automated mapping of the 3D protein structure to the relative positions of its DPD backbone particles which supports realistic 3D protein start geometries in the simulation box.

The SPICES related polymer construction exploits the syntax characteristics of the SPICES definition [25] and is realized by an interplay of the SPICES monomer editor (feature *Monomer definition*) and the SPICES structure editor (feature *Molecule definition*) for polymer construction from monomers.

**Fig. 6:**
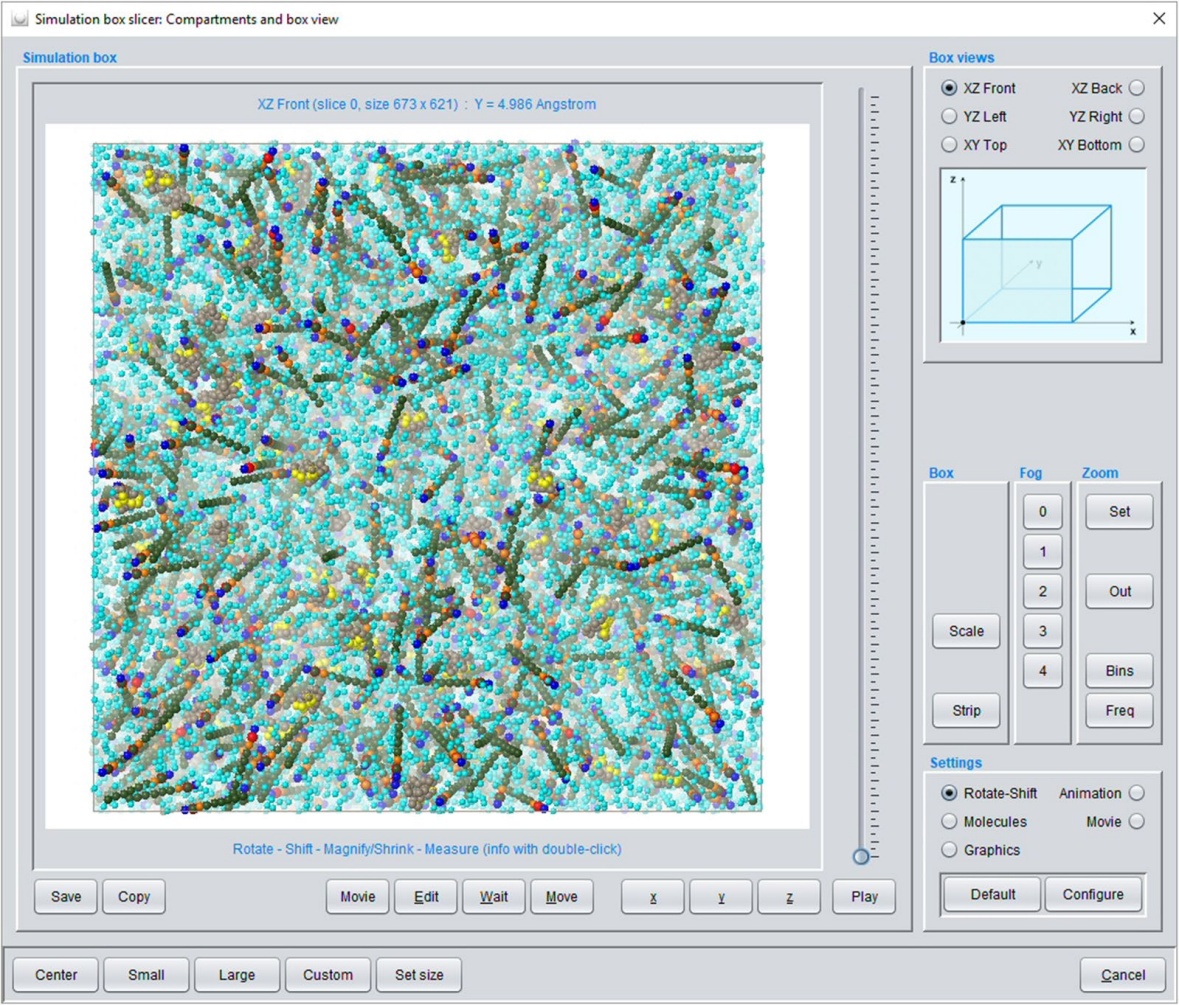
Default random simulation box start configuration without compartment definition (compare Fig. 7) of a chemical ensemble that comprises 800.000 (bulk) water particles (excluded from display), 40.000 water particles (to be located inside a vesicle, colored cyan), 2.200 (oriented outer-vesicle) DMPC molecules (with SPICES line notation: TriMeNP[START]-DMPN(MeAc-6Et)(MeAc-6Et[END]) [25]), 1.800 (oriented inner-vesicle) DMPC molecules (with SPICES line notation: TriMeNP[END]-DMPN(MeAc-6Et)(MeAc-6Et[START]) [25]) and 500 Kalata B1 cyclotides. All molecules—except the Kalata B1 cyclotides which are derived from PDB file 1NB1 [47]–are generated as randomly-oriented spatial SPICES 3D tubes at random positions in the box. The 500 Kalata B1 cyclotides are also randomly distributed throughout the box but their individual amino acid backbone particle positions are set according to their spatial 3D structure (with the side chain particles collapsed onto their neighboring backbone particles). In addition, each Kalata B1 cyclotide is overall shrunk into a virtual sphere (with a volume that corresponds to its particles’ DPD volume) from which all other particles are excluded–thus a Kalata B1 cyclotide start size is somewhat smaller than its actual size during simulation. DMPC particle colors: DMPN (red), Et (olive), MeAc (orange), TriMeNP (blue). Kalata B1 backbone particles are shown in beige (all amino acid side chain particles are excluded from display) with the backbone particles that correspond to the characteristic hydrophobic spot of these peptides displayed in yellow

**Fig. 7:**
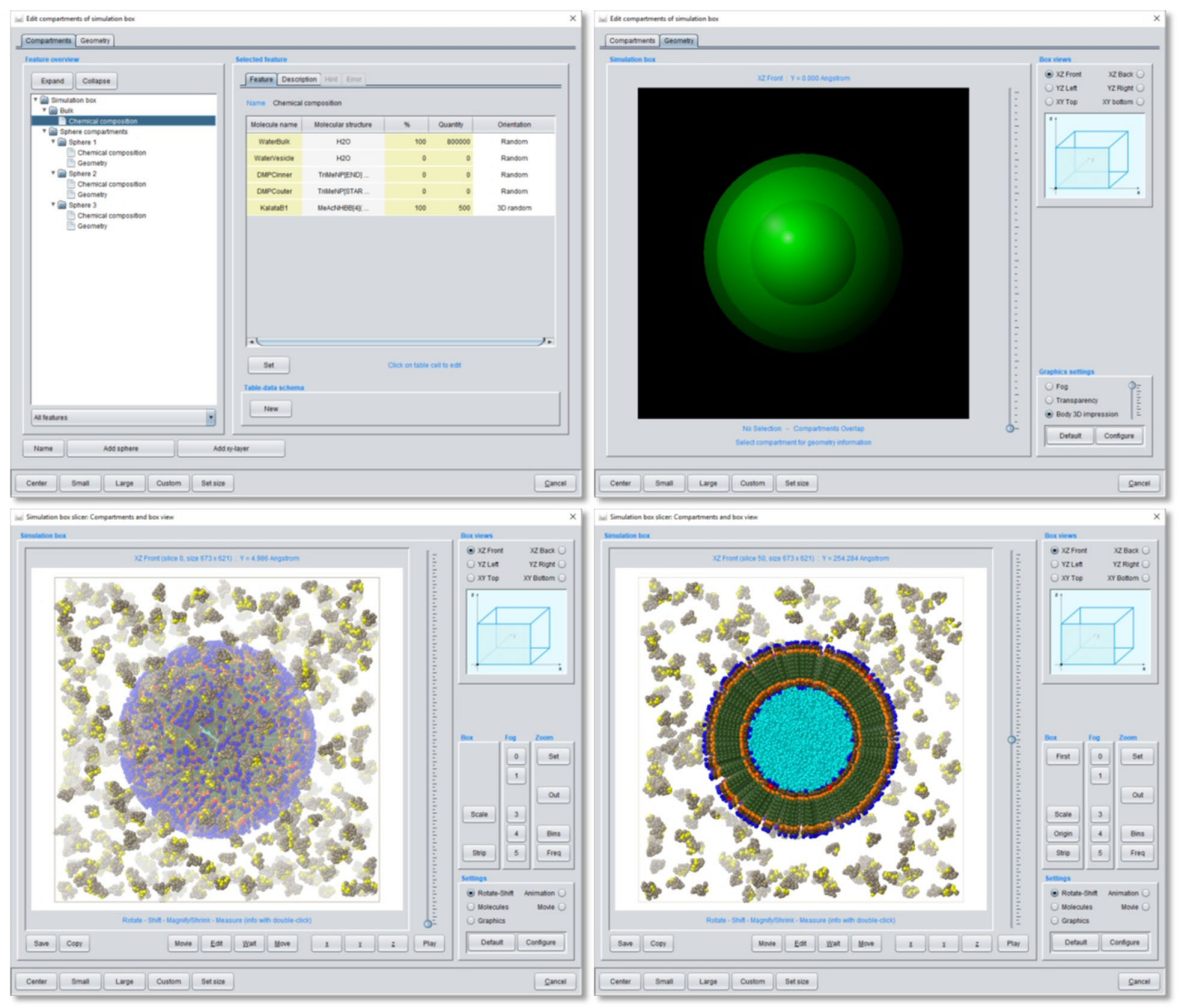
Definition of compartments for a 30 nm DMPC phospholipid double-layer vesicle and view of the resulting simulation box start configuration of the chemical ensemble described in Fig. 6. Upper left: Modal compartment editor dialog with detailed settings of the bulk phase (with all Kalata B1 cyclotides and the 800.000 bulk water particles) outside any compartment and the 3 sphere compartments for oriented spatial positioning of the inner/outer DMPC phospholipids and the inner vesicle water particles. Upper right: Graphical display of size and spatial position of the 3 overlaid sphere compartments. Lower left: Corresponding *Simulation box slicer* view of the simulation box start configuration. Lower right: Cross-section through the simulation box for a more detailed view of the DMPC molecule orientation inside the vesicle

The *Simulation box* subsection of the *Chemical system description* (see Fig. 3 for the expanded feature tree) provides detailed setting options for the molecular ensemble. The *Composition* sub-subsection allows the definition of the number of molecules in the box where *Concentration* settings in *gram*, *mol*, *mol*-*percent* or *weight*-*percent* are also possible. Feature *Box size* may modify the box geometry with a cube as a default. The *Colors* sub-subsection defines different color display modes with *Molecule*-*particle color* as the most fine-grained setting where an individual color may be chosen for every particle type within a molecule. The *Particles and molecules display* sub-subsection then allows for the corresponding individual default display settings for all molecules/particles (which may be arbitrarily changed at a later stage). The generated default molecular simulation box configuration consists of randomly-oriented spatial SPICES 3D tubes (described in [25]) at random box positions (see Fig. 6 which also describes the treatment of PDB-derived 3D peptide/protein structures). The *Compartments and box view* feature (selected in Fig. 3) may be used to set up specific compartments with specific molecule orientations and to view the resulting start configuration of the simulation box. The compartment editor enables various settings as a combination of sphere, layer or rectangular cuboid compartments where different molecular orientations may be defined within each compartment (see Fig. 7). Layers with exclusively single particle molecules allow for a simple cubic lattice particle positioning which may generate a solid surface in combination with the *Molecule fixation* function (see below). The editor also supports a correct a priori DPD density of particles inside a compartment (which may be arbitrarily changed).

The settings of the final *Movement* sub-subsection of the *Simulation box* subsection address molecule movement control capabilities of the Jdpd simulation kernel: Molecules may be spatially fixed (*Molecule fixation*) or their movement restricted to virtual cages surrounded by “reflective walls” within the simulation box (*Molecule boundary*). On the other hand, molecules may possess a fixed velocity (*Molecule fixed velocity*) or may be kicked with a specified frequency (*Molecule acceleration*).

The final *Property calculation* subsection of the *Chemical system description* offers several calculation options during simulations like particle-pair radial distribution functions (RDF) and distances, particle-based molecular radii of gyration or a detailed particle/molecule nearest-neighbor analysis to monitor changing particle/molecule vicinities with temporal evolution of the simulated ensemble.

*Interaction description* as the third main *Job Input* section addresses the fundamental physics of the DPD particle–particle interactions like the *Temperature* setting, *Random DPD force magnitude*, electrostatic interactions of charged particles, gravitational acceleration or the characteristics of bonds between particles and their neighbor particles within molecules. Since mesoscopic DPD simulations are based on isotropic particle–particle repulsions (editable via feature *Particle interactions* with the default repulsion parameters taken from the selected particle set) it may be necessary to impose preferential molecular conformations, e.g. to stabilize the 3D backbone structure of proteins by adequate spring forces between backbone particles to prevent a structural collapse. Thus, additional particle–particle spring forces may be specifically defined between indexed particles in SPICES line notations (*Molecule backbone forces*), the amino acid backbone particles of peptides and proteins (*Protein backbone forces*) and the backbone particles of peptides and proteins within assigned chain segments (*Protein distance forces*).

The final main *Simulation description* section completes the *Job Input* settings with definitions for control of the simulation itself. The number of *Simulation steps* defines the temporal physical period to be simulated by integration of the equations of motion with a defined *Time step length* and a specific *Integration type*. *Output frequency* controls the intermediate output (e.g. the creation of particle position files for specific simulation steps) for later evaluation of the simulation record. *Initial minimization steps* may be defined to improve the initial simulation box start configuration with *Minimization step output* as a control setting for later inspection. Remaining definitions address *Periodic boundaries* along the box axes, the use of a *DPD unit mass*, possible *Initial velocity scaling steps* for temperature control and the choice of the *Random number generator* for the random DPD force.

A completely defined job may be finally saved (*Apply* button of the modal *Job Input* dialog) with an automated addition to the list of available *Job Input* definitions of the current workspace.

### Simulation job execution

The *Execution* tab of the basic GUI frame manages the simulation of defined (error-free) *Job Input* definitions and a restart of already simulated *Job Result* instances (with a defined number of additional simulation steps): After addition to the *Job Execution Queue* jobs may be executed in parallel according to the global settings defined in *Preferences/Parallel computing*. For job execution MFsim must convert a *Job Input* definition to a Jdpd command file with corresponding particle position files (see class *JobUtilityMethods* in package *model.job* as well as Appendix 3). Completed simulations are stored as new *Job Result* instances for further evaluation (see below). Job execution is always performed in background so it does not interfere with the concurrent usage of any other MFsim function (with the exception that a currently simulated *Job Input* definition is not allowed to be changed by editing operations or removal). For every job in simulation its progress in percent and the estimated remaining simulation period is displayed and constantly updated. The *Job Execution Queue* may be altered in an arbitrary manner after execution start where a job in simulation cannot be immediately killed but only stopped with a delay to guarantee a valid *Job Result*. All *Job Result* evaluation functions are also available for jobs in execution which allows for a detailed inspection and analysis of simulation progress.

The *Job Result* restart feature offers the possibility of successive simulation pipelines since underlying *Job Input* definitions may be changed before a restart. As an example, a possible molecule fixation or caging defined in the *Movement* sub-subsection (see above) could be removed before a restart of an initial relaxation simulation to allow for new modes of interaction between the molecular species. Changes of the molecular composition or the spatial configuration are not possible since they would affect the overall physical state of a simulation.

### Simulation job result evaluation

The *Results* tab of the basic GUI frame comprises a list of all *Job Result* instances of the current workspace where a *Job Result* may be viewed, removed, imported from or exported to an archive file.

**Fig. 8:**
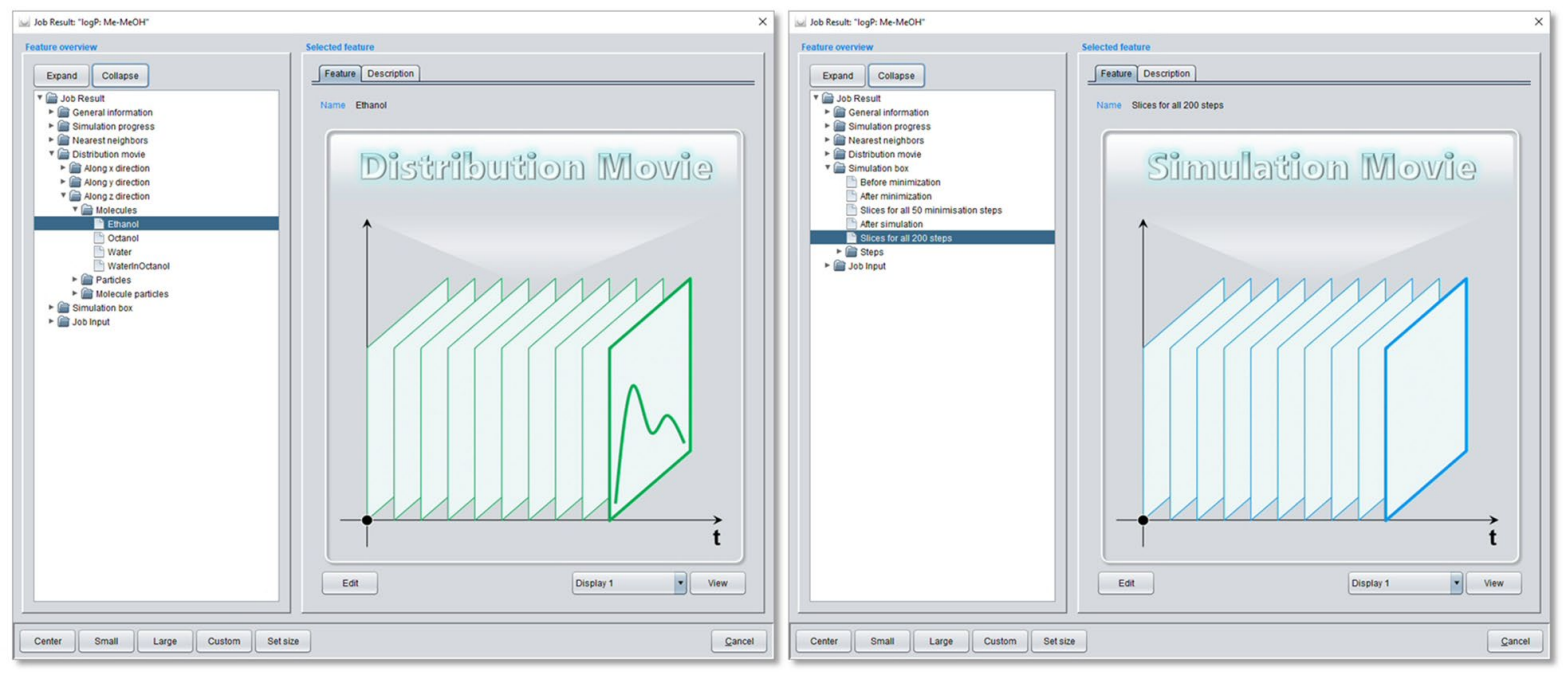
Feature tree of the modal *View* dialog of a *Job Result* with selected *Distribution Movie* feature (left) and *Simulation Movie* feature (right)

The *View* of a *Job Result* opens a modal dialog that consists of a feature tree which offers substantial simulation related evaluations (see Fig. 8). The *General information* section summarizes general settings and results like simulation end point and period, number of simulation steps and corresponding physical time, parallelization settings or the used particle set file name. The *Simulation progress* section provides time-step data and corresponding graphical 2D diagram charts of relevant physical quantities like temperature, kinetic and potential energy or surface tension. The 2D diagram charts may be converted to movies that are in concordance with simulation movies (see below) to allow for a combined display. If specific property calculations throughout the simulation were defined in the *Property calculation* subsection of the underlying *Job Input* definition the corresponding result evaluations appear as additional sections in the *Job Result* feature tree, e.g. the *Nearest neighbor* section in Fig. 8. The *Distribution movie* section (see Fig. 8) provides movies with animated 2D diagram charts of a particle/molecule frequency along a selected axis for a defined simulation period.

**Fig. 9:**
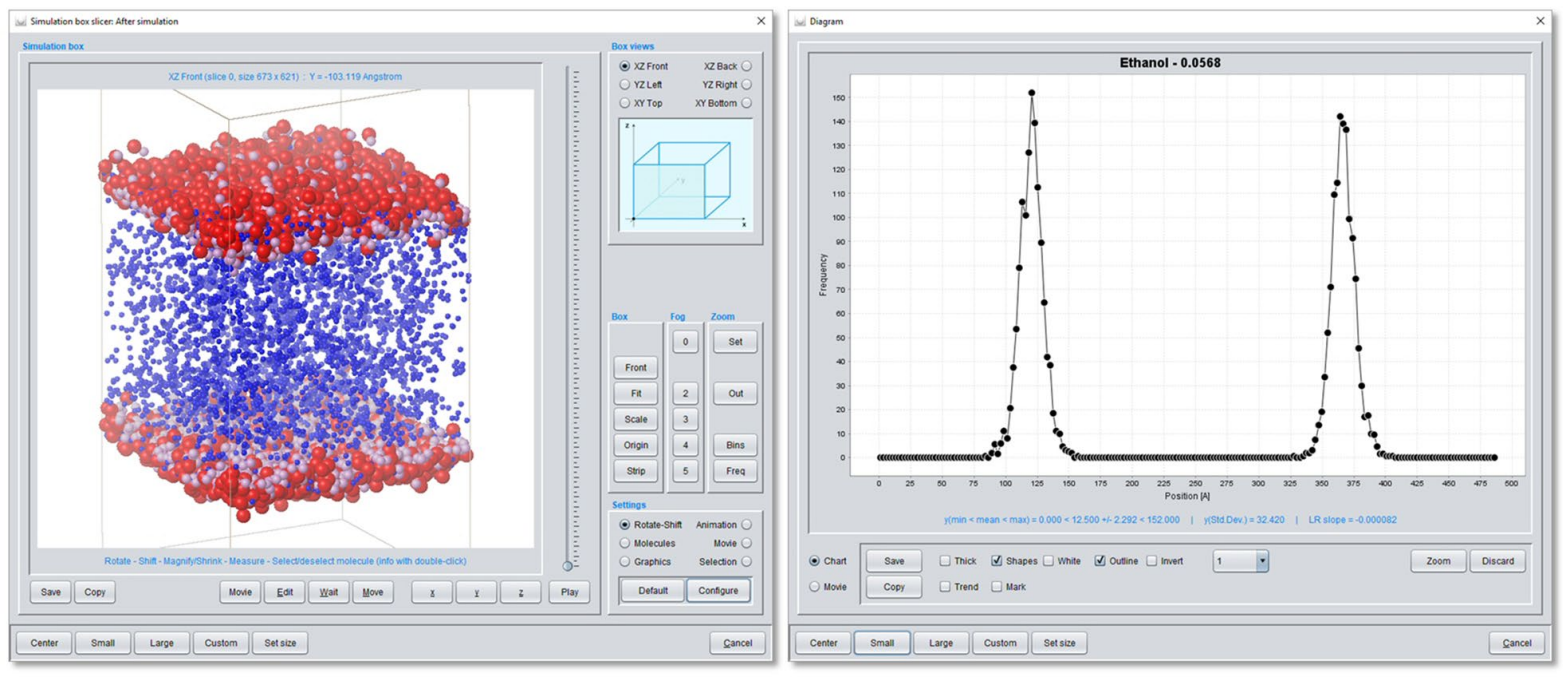
*Simulation box slicer* display of ethanol molecules (SPICES line notation: Me-MeOH) in a simulations box. The diagram on the right quantifies the ethanol frequencies along the z-axis with two distinct layers and corresponds to the ethanol distribution in the simulation box on the left. Ethanol particle colors: Me (plum), MeOH (red). All other molecules of the ensemble are discarded from display except water particles (blue) between the layers

The *Simulation box* section provides (animated) simulation box views of all (output) steps of the simulation process including the a priori minimization steps (if defined in the *Job Input* definition) and the generation of simulation movies for defined simulation periods (see Fig. 8). For simulation box view and analysis MFsim consists of two types of viewers for arbitrarily rotated or shifted box inspections: The *Simulation box viewer* (mis)uses the graphical Jmol capabilities designed for atom-based molecule representations to display particle structures whereas the *Simulation box slicer* generates successive slice images of the simulation box using Java2D. Moreover, the *Simulation box slicer* provides extensive box analysis features like through-space measurements, (individual) particle selections or the definition of zoom volumes with a corresponding particle/molecule frequency analysis (see Fig. 9). Molecular color, size, visibility or transparency settings may be arbitrarily changed for alternative views of the molecular ensemble in question.

Simulation movies for a defined simulation period are generated with the slicer functionality. Slicer related animation settings (e.g. box spinning, box slicing or box zooming) may be used to contribute to the movie generation in an additive manner: A simulation movie may start with a spinning box, then show the minimization steps, again stop with a spinning box after minimization, exclude specific particles/molecules, zoom-in, then show a defined part of the simulation process within the zoomed box volume etc. All slicer functions produce sequences of images which may finally be merged into a MP4 movie clip with the open FFmpeg software (automated integration of FFmpeg is currently only available for the Windows OS).

Last but not least the final *Job Input* section just shows all features of the underlying *Job Input* definition.

### MFsim use cases

The new MFsim environment may be beneficial for different fields of research. Theoretical physicists and chemists that contribute to the foundations of mesoscopic simulation with new methods/models/applications or improved/extended particle parameter sets may choose the open environment for an exemplification of their work so that their new insights and developments can be more easily adapted and utilized by a wider scientific community where especially scientific end-user communities appreciate availability within a rich-client system.

Chemo- and bioinformaticians may likewise be interested in the extended possibilities to popularize their new and improved algorithmic solutions by offering a versatile usage in a convenient environment with only comparatively small additional development efforts.

As an open all-in-one rich-client system with (modest) hardware requirements that are commonly available in scientific institutions, MFsim especially targets scientific end-users without programming skills. Presumed, a theoretically sound simulation model is available, specific alterations and extensions can be performed by end-users themselves. Tutorials *Simulation of a DMPC bilayer membrane model* [48, 49] and *Cyclotide*-*membrane sandwich interaction model* [50, 51] illustrate details of biomolecular simulation setups in a step-by-step fashion (with the corresponding MFsim Job Inputs being available in the MFsim GitHub repository tutorials subfolder) to show that the inevitable training hurdle for productive usage is manageable with tolerable effort. The MFsim GitHub repository also contains links to MFsim generated simulation clips (see README in subfolder *2020 Cyclotide*-*membrane interaction study*) which demonstrate an especially attractive feature for visual communication of research findings (the convenient MFsim support for simulation movie generation is to be outlined in detail in an upcoming tutorial).

In summary, MFsim may broaden the applicability of mesoscopic approaches (at best in close collaboration with experimental research) and stimulate the collaboration between the different disciplines from theory over computing to end-user application.

## Conclusions

With the MFsim project an open simulation environment for mesoscopic simulation is provided with the default Jdpd simulation kernel for Dissipative Particle Dynamics. MFsim supports polymer and especially biomolecular simulations containing peptides and proteins aiming at pushing the mesoscopic simulation frontiers towards these areas of research which often require the study of large systems on the microsecond scale. As an open rich-client all-in-one approach MFsim targets theoretical, computational as well as end-user scientists (without programming skills) thus it contributes to making computational tools more widespread in the scientific community. For the chemo- or bioinformatician the project may serve as a starting point for specific customizations where the reusable functionality may outweigh the initial training hurdle.

MFsim is publicly available as open source published under the GNU General Public License version 3 [52]. The MFsim GitHub repository contains all Java bytecode libraries, a Windows OS installer and a corresponding installation tutorial [53, 54], Javadoc HTML documentations [55] and the Netbeans [56] source code packages including Unit tests. A growing number of tutorials that outline more specific features and use-cases as well as an MFsim based biomolecular research study concerning the interaction of cyclotides and bilayer membranes are in preparation.

## Data Availability

MFsim repository at https://github.com/zielesny/MFsim. Project name: MFsim, Project home page: MFsim repository at https://github.com/zielesny/MFsim, Operating system(s): Platform independent (but automated FFmpeg integration currently only on Windows OS), Programming language: Java, Other requirements: Java 1.8 or higher, open libraries: Apache Commons IO [33] /Lang [34] /RNG [35], BioJava [36, 37], FFmpeg (currently with the Windows OS only), GraphStream [38], Jama [39], JCommon [40], Jdpd, JDOM [41], JFreeChart [42], Jmol [43], PCG [44, 45], SPICES [26] and Vecmath [46]., License: GNU General Public License version 3.

## Appendix 1—Conversion formulas

The appendix comprises conversion formulas between physical and DPD units [5, 6, 57] as well as formulas for molecule concentration related calculations implemented in MFsim.

The conversion between DPD lengths and physical lengths is based on the conversion radius $r_{c}$ (“radius of interaction”) in physical units

$$\begin{aligned} r_{c} = \sqrt[3]{{V_{\hbox{min} } \;\rho_{DPD} \frac{{\sum\limits_{i = 1}^{{N_{particles} }} {N_{particle,i} \frac{{V_{particle,i} }}{{V_{\hbox{min} } }}} }}{{\sum\limits_{i = 1}^{{N_{particles} }} {N_{particle,i} } }}}} \\ \\ l_{phys} = l_{DPD} \;r_{c} \\ \end{aligned}$$

$V_{\hbox{min} } ,$ volume of smallest particle in physical units; $\rho_{DPD} ,$ DPD (number) density; $N_{particles} ,$ number of different particle types; $N_{particle,i} ,$ number of particles of type i; $V_{particle,i} ,$ volume of particle of type i in physical units; $l_{phys} ,$ length in physical units; $l_{DPD} ,$ length in DPD units.

with the conversion between DPD time and physical time being approximated by

$$t_{phys} = t_{DPD} \;f_{soft} \;r_{c} \;\sqrt {\frac{1}{R\;T}\frac{{\sum\limits_{i = 1}^{{N_{particles} }} {N_{particle,i} \;M_{particle,i} } }}{{\sum\limits_{i = 1}^{{N_{particles} }} {N_{particle,i} } }}}$$

$t_{phys} ,$ time in physical units; $t_{DPD} ,$ time in DPD units; $f_{soft} ,$ factor for increased particle diffusivity due to soft potentials ($f_{soft} \approx 1000$); $R,$ gas constant; $T,$ thermodynamic temperature; $M_{particle,i} ,$ molar mass of particle of type i.

Molecule concentration calculations are based on the relations

$$N_{molecule,1}^{scaled} = \frac{{\rho_{DPD} V_{box,DPD} }}{{\sum\limits_{k = 1}^{{N_{particles} }} {N_{1k} } + \sum\limits_{i = 2}^{{N_{molecules} }} {\left( {\frac{{n_{molecule,i}^{scaled} }}{{n_{molecule,1}^{scaled} }}\sum\limits_{k = 1}^{{N_{particles} }} {N_{ik} } } \right)} }}{ ; } N_{molecule,i \ne 1}^{scaled} = N_{molecule,1}^{scaled} \frac{{n_{molecule,i \ne 1}^{scaled} }}{{n_{molecule,1}^{scaled} }}$$

$$n_{molecule,i}^{scaled} = \frac{{n_{molecule,i} \;s_{molecule,i} }}{{\sum\limits_{j = 1}^{{N_{molecules} }} {n_{molecule,j} \;s_{molecule,j} } }}$$

$$s_{molecule,i} = \frac{{\sum\limits_{k = 1}^{{N_{particles} }} {N_{ik} \frac{{V_{particle,k} }}{{V_{\hbox{min} } }}} }}{{\sum\limits_{k = 1}^{{N_{particles} }} {N_{ik} } }}$$

$$n_{molecule,i} = \frac{{\frac{{w_{molecule,i} }}{{\sum\limits_{k = 1}^{{N_{particles} }} {N_{ik} \;M_{particle,k} } }}}}{{\sum\limits_{j = 1}^{{N_{molecules} }} {\left( {\frac{{w_{molecule,j} }}{{\sum\limits_{k = 1}^{{N_{particles} }} {N_{ik} \;M_{particle,k} } }}} \right)} }}$$

$$w_{molecule,i} = \frac{{N_{molecule,i} \sum\limits_{k = 1}^{{N_{particles} }} {N_{ik} \;M_{particle,k} } }}{{\sum\limits_{j = 1}^{{N_{molecules} }} {\left( {N_{molecule,j} \sum\limits_{k = 1}^{{N_{particles} }} {N_{jk} \;M_{particle,k} } } \right)} }}$$

$N_{molecule,i}^{scaled} ,$ Number of molecules of type i in simulation box; $V_{box,DPD} ,$ volume of simulation box in DPD units; $N_{ik} ,$ number of particles of type k in single molecule of type i; $N_{molecules} ,$ number of different molecule types; $n_{molecule,i}^{scaled} ,$ volume-scaled relative number of molecules of type i; $n_{molecule,i} ,$ relative number of molecules of type i; $s_{molecule,i} ,$ volume scaling factor of molecule of type i; $w_{molecule,i} ,$ relative weight of molecule of type i; $N_{molecule,i} ,$ number of molecules of type i.

## Appendix 2—Data objects, preferences and re-usable settings

All data items for GUI display are stored in *ValueItem* objects (package *model.valueItem*) which can be configured for data structures like scalars, vectors or matrices (see enumeration *ValueItemEnumBasicType* in package *model*.*valueItem*) that consist of basic data types like texts, numbers, directory paths or links to specific dialogs (see enumeration *ValueItemEnumDataType* in package *model*.*valueItem*). A *ValueItem* object may contain default values as well as specific data checks like min/max boundaries for numeric values, selection texts or regular expressions for control of textual input in order to prevent input errors. Related *ValueItem* objects may be encapsulated by a *ValueItemContainer* object (package *model*.*valueItem*) which itself may be used for a tree view of its contents via GUI display. Moreover, a *ValueItemContainer* object allows for an interplay of its *ValueItem* objects like directed update cascades after value changes (e.g. see class *UtilityJobUpdate* in package *model*.*job* for update cascades concerning *Job Input* definitions). *ValueItem* and *ValueItemContainer* objects can be made persistent via XML serialization.

Global preferences are realized by a modifiable *Preferences* singleton (package *model*.*preference*) which provides adequate *ValueItem* objects for all settings. The global preferences dialog is available via menu entry *Application/Preferences/Edit* of the basic GUI frame.

A particular feature for efficient usage is the implemented table-data schema management. A table-data schema is a pattern of values for vectors or matrices of *ValueItem* objects which can be named and internally stored for reuse: Schemata alleviate complex setting operations e.g. for the graphical display of multiple particles. A table-data schema manager is available via menu entry *Application/Schemata/Manage* of the basic GUI frame and provides view, edit, remove or clear functions with an additional file export of schemata lists for exchange. Persistent schemata files may be reloaded or merged.

The definition of *ValueItem* related vectors or matrices is supported by bulk functions which can be refined by column-based filters. Thus, the tedious input of multiple values (e.g. for force constants or colors) may be realized by a few manual operations.

## Appendix 3—Simulation kernel integration

Jdpd is integrated as a command text file driven simulation kernel: All settings are comprised in an *Input.txt* text file with references to compound related *PositionsBonds *< *index *> *.txt* text files which contain all initial particle xyz positions and particle–particle spring-force definitions for all molecular species. During simulation Jdpd produces (partly compressed) output text files in form of simple xy table data for e.g. relevant physical quantities versus simulation step (like *T.txt* for temperature, *UpotDpd.txt* for conservative DPD energy etc.), particle position files which provide all particle positions at a specific simulation step in the simulation box (e.g. *PP2400.gz* for particle positions of simulation step 2400) as well as structurally more complex text files like *M_M_TUPLE.txt* for nearest-neighbor data. Thus, an integration of Jdpd requires the automated generation of the necessary input text files, the programmatic control of Jdpd during simulation (with intermediate simulation progress inspections or possible exception/error handling) and the automated analysis of the simulation record contained in the Jdpd generated output text files: The code for these tasks is mainly located in package *model.job* for input/output text file generation/analysis and in class *MainFrameController* (section *Job execution related command methods*) in package *gui.main* for Jdpd control during simulation.

Class *JdpdValueItemDefinition* (package *model.job*) defines in method *initializeJdpdValueItems()* the *ValueItem* instances for all job input settings (MFsim stores data in *ValueItem* objects, see previous appendix) which are collected in a *ValueItemContainer* instance. This job input *ValueItemContainer* instance is then modified during job design with a cascaded top-down interplay of the *ValueItem* instances coded in class *JobUpdateUtils* (package *model.job*) to prevent later ill-defined settings. A completed job design results in a corresponding job input *ValueItemContainer* instance (which is then made persistent via XML file output). Before instantiation of a Jdpd kernel instance the information of a job input *ValueItemContainer* instance is parsed to Jdpd input and position/bonds text files via method *getJdpdInputText()* of class *JobUtilityMethods* (package *model.job*). Then a Jdpd kernel instance is created with the generated text files as its main input and submitted to a thread-pool for concurrent execution (methods *startJobsInJobExecutionQueue()* and *startRemainingJobExecutionTasks()* in class *MainFrameController*, package *gui.main*). During as well as after simulation the Jdpd generated simulation record is mapped back to corresponding *ValueItem* instances (collected in a job result *ValueItemContainer* instance) via method *getResultValueItemContainerForJobResult()* of class *JobResult* (package *model.job*) which are visualized by MFsim for job result analysis.

To integrate an alternative simulation kernel software the sketched classes and methods have to be customized accordingly. In addition, the kernel control structure has to be adjusted for polyglot programming since scientific kernel software is usually written in languages like C/C ++ or FORTRAN and ahead-of-time compiled to executables. To realize the integration task the elaborated capabilities of the Java platform can be utilized. Thus, from a software development point of view, the integration into the MFsim environment allows for an extremely flexible response to a wide range of requirements – where the existing Jdpd integration code may serve as a productive blueprint. It can be estimated from experience with previous integration tasks that—a skilled software developer presumed—a simulation kernel integration requires at least several weeks (for comparatively simple and less demanding solutions) up to several months (for more complex, comprehensive and thoroughly safe-guarded solutions like the Jdpd integration).
